# Microbial intervention by *Acinetobacter schindleri* SR-5-1 alleviates cadmium toxicity in pea cultivation

**DOI:** 10.1080/15592324.2026.2689808

**Published:** 2026-06-24

**Authors:** Sherjeel Hashmat, Muhammad Tariq Javed, Umer Farooq, Hossam S. El-Beltagi, Usman Zulfiqar, Mohammed S. Alotaibi, Nazih Y. Rebouh, Mayank Anand Gururani

**Affiliations:** a Department of Botany, Faculty of Life Sciences, Government College University, Faisalabad, Pakistan; b Agricultural Biotechnology Department, College of Agriculture and Food Sciences, King Faisal University, Al Ahsa, Saudi Arabia; c Department of Agronomy, Faculty of Agriculture and Environment, The Islamia University of Bahawalpur, Bahawalpur, Pakistan; d Department of Biology, Nakhchivan State University, Nakhchivan, Azerbaijan; e Department of Clinical Laboratories Sciences, Tarabah University College, Taif University, Taif, Saudi Arabia; f Institute of Environmental Engineering, RUDN University, Miklukho-Maklaya St., Moscow, Russia; g Department of Biology, College of Science, United Arab Emirates University, Al Ain, United Arab Emirates

**Keywords:** Antioxidants, cadmium stress, growth, nutrient profile, oxidative injury, pea, *Acinetobacter schindleri* SR-5-1

## Abstract

Cadmium (Cd) contamination of agricultural soils disrupts plant signaling networks, impairing nutrient communication, photosynthetic efficiency, and stress responses. Microbial inoculants offer eco-biotechnological solutions by modulating signal perception and transduction under heavy metal stress. This field study investigated the role of *Acinetobacter schindleri* strain SR-5-1 in influencing pea (*Pisum sativum* L.) signaling pathways under Cd toxicity. Plants exposed to environmentally relevant Cd concentrations (250 and 500 µM) exhibited disrupted chlorophyll biosynthesis, elevated oxidative stress markers, and impaired nutrient signaling. Inoculation with SR-5-1 restored chlorophyll levels, enhanced ROS-scavenging enzyme activities, and reduced lipid peroxidation, indicating microbial effects on oxidative signaling cascades. Importantly, the inoculated plants accumulated less Cd in the roots and leaves, reflecting microbial mediation of ion transporter activity and rhizosphere detoxification. SR-5-1 also improved nitrogen, iron, zinc, potassium, and magnesium acquisition, highlighting its role in nutrient uptake and homeostasis. These findings demonstrate that SR-5-1 functions as a bio-communicator, alleviating xenobiotic stress by modulating ROS and nutrient signaling pathways. The study underscores the ecological relevance of microbial inoculants in supporting integrative plant communication and resilience, positioning SR-5-1 as a promising bioresource for signaling-driven sustainable agriculture in Cd-affected soils.

## Introduction

Cadmium (Cd) is a highly toxic, non-essential heavy metal that poses a significant threat to global plant health due to its existence in the environment and has a high ability to move in soils and water systems.^1^ Its sources include industrial emissions, mining, use of phosphate fertilizer, sewage sludge, and poor agricultural practices.^2^ Cadmium contamination is a widespread environmental problem, impacting both agricultural productivity and food safety.^3^ Pakistan, like many developing countries, faces challenges with Cd contamination in agricultural soil due to rapid industrialization, use of untreated wastewater for irrigation, and overuse of fertilizer.^4^ Studies conducted in Pakistan have demonstrated that Cd is extremely toxic to major crops, including wheat.^5^ Even at low concentrations (5 mg/L), Cd significantly inhibits root and shoot growth, while higher concentrations (20-80 mg/L) drastically reduce seed germination and seedling vigor.^6^ The reduction in crop growth and yield due to Cd not only threatens food security but also increases the harm of Cd entering the human food chain by consumption of contaminated grains and vegetables.^7^ The accumulation and translocation of essential minerals are disrupted, which causes nutrient disparities and further physiological stress.^7^

Cadmium stress poses a significant challenge for plants, negatively impacting their ability to produce photosynthetic pigments, which are crucial for photosynthesis and overall growth.^8^ Higher levels of Cd consistently reduce the content of chlorophyll pigments in plants,^9^ primarily due to Cd interference with the enzymes responsible for its production, such as protochlorophyllide reductase and the synthesis of 5-aminolevulinic acid.^10^ Additionally, Cd can disrupt the chlorophyll molecule by replacing magnesium and forming inactive complexes, which further hampers the plant's capacity to photosynthesize effectively.^11^ Plants experience oxidative stress under Cd toxicity due to the genesis of reactive oxygen species (ROS), which causes a detrimental impact on chloroplast structures and vital proteins involved in photosynthesis, particularly photosystem II (PSII).^12^ Such damage can be observed through decreased chlorophyll fluorescence and reduced efficiency in the electron transport process, ultimately lowering the plant's photosynthetic effectiveness.^13^ Interestingly, chlorophyll *b* tends to be more sensitive to Cd toxicity compared to chlorophyll *a*, resulting in altered ratios of these pigments.^14^ Consequently, plants experience a decline in critical gas exchange parameters, such as their net photosynthetic rate and stomatal conductance, which impacts their ability to utilize light effectively.^15^ Research on a variety of plants, including lettuce,^16^ borage,^17^ *Artemisia annua*, ^18^ and hybrid Pennisetum,^19^ has corroborated these observations, highlighting symptoms like leaf yellowing, chlorosis, and decreased pigment levels when subjected to cadmium stress. The primary way in which Cd affects photosynthetic pigments involves hindering their production, damaging chloroplasts, and disrupting the electron transport process.^20^

Exposure to Cd sets off a series of metabolic reactions in plant cells that result in elevated lipid peroxidation and excessive ROS production.^21^ By interfering with the antioxidant defence system and creating an imbalance between ROS generation and scavenging, Cd upsets the cellular redox equilibrium. ROS, such as hydroxyl radicals, superoxide anions, and hydrogen peroxide, build up because of this imbalance and target the polyunsaturated fatty acids found in membrane lipids.^21^ This lipid peroxidation weakens the fluidity and integrity of the membrane, which allows the cell contents to seep out and reduces the activity of enzymes that are membrane-bound.^22^ Malondialdehyde (MDA), one of the byproducts of lipid peroxidation, is a biomarker of oxidative damage and contributes to the spread of oxidative stress by decreasing the synthesis of proteins and nucleic acids.^23^ Furthermore, the hexose monophosphate pathway is disrupted by Cd, which reduces the generation of NADPH and hinders the body's ability to detoxify ROS^24^. The degree of oxidative damage is reflected in ultrastructural alterations such as thylakoid dilatation and disarray of the chloroplast membrane.^25^ The toxic effects of cadmium stress in plants are instigated by these pathways, which, taken together, result in cellular dysfunction and emphasize the crucial roles that oxidative stress and lipid peroxidation play in Cd-induced phytotoxicity.^26^

Through several intricate processes, Cd interferes with the absorption and homeostasis of vital nutrients, including magnesium (Mg), zinc (Zn), potassium (K), iron (Fe), phosphorus (P), and nitrogen (N).^27^ Because of its comparable ionic radius, which competitively inhibits the absorption of divalent cations, including Fe²⁺, Ca²⁺, Mg²⁺, and Zn²⁺, Cd mostly enters plant roots via transporters for these cations.^28^ For instance, Cd inhibits Fe (III) reductase activity and competes with Fe at iron transporter IRT1, resulting in a deficit in shoots and an accumulation of Fe in roots, which hinders photosynthesis and chlorophyll production.^29^ Likewise, Cd disrupts enzyme activity and chlorophyll production by interfering with the intake of magnesium and zinc.^30^ Permeability of plasma membranes was altered by Cd toxicity, which results in nutritional loss and hinders transport mechanisms.^31^

*A.* s*chindleri* SR-5-1, a nonmotile and gram-negative bacterium, is widely recognized to perform as plant growth bacterium with bioremediation potential particularly when subjected to Cd stress.^32^ Through processes including nitrogen fixation and metal bioaccumulation, it has been demonstrated to improve nutrient absorption and reduce cadmium toxicity, hence boosting the growth and physiological performance of pea (*Pisum sativum*) plants.^32^ By regulating the activities of antioxidant enzymes, this strain can lessen oxidative stress in plants and shield cellular structures from harm caused by cadmium.^33^ Furthermore, *A.* s*chindleri* SR-5-1 encourages morphological and biochemical changes in pea and linseed plants, which enhances their resistance to Cd wastewater stress and improves their general health.^32^ Its potential as a bioinoculant for sustainable agriculture in polluted soils is shown by its use in pea growing under cadmium stress.

The problem statement for this study arises from the critical need to mitigate cadmium (Cd) toxicity in agricultural soils, which severely impairs pea (*Pisum sativum*) growth and productivity. Although previous pot experiments demonstrated that inoculation with *A. schindleri* SR-5-1 can reduce Cd stress by enhancing plant growth and reducing Cd accumulation, it remains essential to validate these findings under realistic field conditions where environmental variables are more complex. The hypothesis is that *A. schindleri* SR-5-1 inoculation will improve pea growth and physiological performance under cadmium toxic dose in the field by promoting nutrient uptake, reducing Cd bioavailability, and enhancing stress tolerance compared to uninoculated controls. The objectives of this field experiment are to (1) evaluate the effects of *A. schindleri* SR-5-1 on pea growth and yield under cadmium stress under field conditions, (2) assess the effects of bacterial inoculation on cadmium absorption in plant tissues, and (3) assess the biochemical responses of pea plants to cadmium toxicity with and without bacterial treatment. This study aims to provide comprehensive highlights for the practical applicability of *A. schindleri* SR-5-1 as a bioinoculant for mitigating heavy metal stress in legumes, thereby contributing to sustainable agriculture and food safety.

## Materials and methods

### Experimental design

A field experiment was conducted to assess the impact of *Acinetobacter schindleri* SR-5-1 inoculation on pea (*Pisum sativum* L.) growth under cadmium (Cd) stress. The study employed a randomized complete block design (RCBD) with four replicates to minimize environmental variability. Each replicate contained 4 plots, resulting in a total of 16 plots (4 treatments × 4 replicates). The plot size was 3 m × 3 m, with 1 m buffer zones between plots and 1.5 m spacing between blocks to reduce edge effects.

### Plant material and seed preparation

Certified pea seeds were obtained from the Ayub Agricultural Research Institute (AARI), Faisalabad, Punjab, Pakistan. Seeds were surface-sterilized by soaking in 1% sodium hypochlorite for 5 min, followed by thorough rinsing with sterile distilled water. Treatments were randomly assigned within each block.

### Bacterial inoculation

#### Acinetobacter schindleri

Strain SR-5-1 was isolated from the rhizosphere soil of pea (*Pisum sativum* L.) cultivated in Cd-contaminated fields in Faisalabad, Pakistan. Selective isolation was performed on nutrient agar medium supplemented with CdCl₂ (250 µM) to enrich for Cd-tolerant colonies. The isolate was purified by repeated streaking and identified based on morphological traits, Gram staining, and biochemical profiling. To evaluate Cd sequestration ability, SR-5-1 was cultured in nutrient broth amended with increasing Cd concentrations (250  µM and 500  µM). Growth performance was monitored by optical density at 600 nm, and Cd sequestration was quantified using atomic absorption spectrophotometry (AAS) after the centrifugation of bacterial cultures. The strain demonstrated tolerance to up to 500  µM Cd and showed significant biosorption capacity, confirming its potential for rhizosphere detoxification and suitability for inoculation trials.

### Soil preparation and fertilization

The experimental site was plowed, harrowed, and leveled to ensure uniform soil conditions. Basal fertilization was applied uniformly to all plots based on soil test recommendations: nitrogen (80 kg/ha), phosphorus (60 kg/ha), and potassium (60 kg/ha). Cadmium stress was imposed by applying cadmium chloride (CdCl₂) solution at the designated concentrations.

### Cadmium stress application

Cadmium stress was imposed using cadmium chloride (CdCl₂) solution. The solution was prepared at concentrations equivalent to 250 and 500  µM Cd, corresponding to environmentally relevant stress levels. The CdCl₂ solution was applied twice during the early vegetative stage to ensure uniform contamination. Each plot received 5 L of CdCl₂ solution per application, evenly distributed across the soil surface. This resulted in final soil Cd concentrations of approximately 120 and 240  mg/kg, as verified by post-application soil analysis.

### Agronomic practices

Standard agronomic practices, including irrigation, weed control, and pest management, were maintained throughout the growing season. The experiment lasted approximately 65 d, corresponding to the full growth cycle of pea plants under local climatic conditions.

### Growth attributes

Plant fresh biomasses were measured using a computerized weight balance. The plants were oven-dried at 75 °C for 3 d to determine dry biomasses.^34^

### Photosynthetic pigments

Photosynthetic pigments, including chlorophyll *a*, chlorophyll *b*, total chlorophylls, and carotenoids, were quantified using spectrophotometric analysis following established protocols. Fresh frozen plant tissue (0.5 g) was homogenized and extracted in 10 mL of 80% acetone for 24 h in darkness. The homogenate was centrifuged at 10,000 × g for 15 min to pellet the cellular debris. The resulting supernatant was carefully collected and analyzed using a Hitachi U-2910 spectrophotometer (Tokyo, Japan). The absorbance readings were taken at 480 nm (carotenoids), 645 nm (chlorophyll *b*), and 663 nm (chlorophyll *a*). Pigment levels were calculated according to the equations defined by,^35^ which enable the determination of individual and total chlorophyll as well as carotenoid content based on the recorded absorbance values. This method ensures precise quantification of photosynthetic pigments while preserving their structural integrity during extraction.

### Oxidative stress indicators

Fresh leaf material (0.5 g) was homogenized in TCA (6%), then centrifuged. The supernatant was added to 50 mM potassium phosphate buffer (0.5 mL; pH 7.8). The reaction mixture was held at room temperature for 30 min. To assess hydrogen peroxide (H_2_O_2_) levels, the reaction mixture's absorbance at 390 nm was measured using a spectrophotometer.^36^

The malondialdehyde (MDA) content was determined according to the method of,^37^ with some modifications. In brief, 0.5 g of fresh leaf tissue was ground in 10 mL of 5% trichloroacetic acid, and the mixture was centrifuged to obtain the supernatant. A portion of this supernatant (0.5 mL) was then combined with 2 mL of 0.5% thiobarbituric acid. The resulting mixture was incubated at 95 °C for 1 h and then cooled rapidly in an ice bath. The absorbance readings were then recorded at 532 and 600 nm using a spectrophotometer to assess the MDA levels.

### Enzymatic antioxidants

Superoxide dismutase (SOD) activity was determined following the method described previously.^38^ The assay mixture, with a total volume of 3  mL, contained 13  mM methionine, 1.3  µM riboflavin, 50 µM nitro-blue tetrazolium (NBT), 75 mM EDTA, and 50 µL of the enzyme extract. This mixture was exposed to fluorescent light for 15 min, alongside a control sample that did not include the enzyme extract. The absorbance of the samples was then measured at 560 nm to evaluate SOD activity.

Peroxidase (POD) activity was assessed following the method described previously.^39^ The reaction mixture, with a total volume of 3 mL, consisted of 40  mM hydrogen peroxide, 20 mM guaiacol, 50 mM potassium phosphate buffer at pH 7.5, and 100 µL of the enzyme extract. The change in absorbance was measured at 470 nm at 20-s intervals over a period of 3 min using a spectrophotometer.

Catalase (CAT) activity was evaluated using a 3 mL reaction mixture containing 5.9  mM hydrogen peroxide, 50  mM potassium phosphate buffer (pH 7.5), and 100  μL of enzyme extract. The reduction in absorbance at 240 nm was recorded every 20 s over a 2-min period. One unit of catalase activity was defined as the amount of enzyme causing a 0.01 decrease in absorbance per minute, according to.^40^

For ascorbate peroxidase (APX) activity, the reaction mixture (1 mL) was prepared by combining 50 mM potassium phosphate buffer (pH 7.5), 0.5 mM ascorbic acid, 0.1 mM hydrogen peroxide, 0.1 mM EDTA, and 100 µL of enzyme extract. The absorbance at 290 nm was measured every 20 s for 2 min using a spectrophotometer. Enzyme activity was expressed in units per milligram of protein, where one unit corresponds to a 0.1 change in absorbance per minute per milligram of protein, as described previously.^41^

### Histochemical analysis

Histochemical analysis was conducted to visualize the spatial distribution of reactive oxygen species (ROS) and lipid peroxidation in pea leaves subjected to cadmium stress with or without *Acinetobacter schindleri* SR-5-1 inoculation. Fresh, fully expanded leaves were collected from each treatment and immediately processed for staining. Hydrogen peroxide (H₂O₂) accumulation was detected using 3,3′-diaminobenzidine (DAB) staining, where the leaves were immersed in 1 mg mL⁻¹ DAB solution (pH 3.8) and incubated under light for 6 h until brown precipitates appeared, indicating the presence of H₂O₂. The stained leaves were subsequently cleared in boiling ethanol (96%) and photographed. Superoxide radicals (O₂⁻) were localized by incubating leaves in 0.1% nitroblue tetrazolium (NBT) solution prepared in 10 mM potassium phosphate buffer (pH 7.8) for 2  h under light, producing blue formazan deposits that marked O₂⁻ accumulation. Lipid peroxidation was visualized using Schiff’s reagent, where leaves were fixed in 0.5% glutaraldehyde, rinsed, and incubated in Schiff’s reagent for 30 min; the appearance of magenta coloration indicated malondialdehyde (MDA) formation. After being stained, the leaves were photographed using a digital microscope, and the intensity and distribution of coloration were compared among the treatments to assess oxidative damage and the protective effect of bacterial inoculation.

### Elemental analysis

To analyze the elemental concentrations in the leaves and roots, dry plant material was subjected to acid digestion following the procedure described by Allen et al.^42^ Phosphorus levels were measured using the spectrophotometric method of Jackson,^43^ while nitrogen content was determined according to Hafez and Mikkelsen.^44^ Potassium concentrations were assessed with a flame photometer (Sherwood, model 410, UK). Leaf and root samples were dried at 70 °C for 1 week, then ground into a fine powder. A 0.1 g portion of the powdered sample was digested with a tri-acid mixture composed of nitric acid, sulfuric acid, and perchloric acid in a 10:1:4 ratio until a clear solution was obtained. The final volume of the digest was adjusted to 25 mL with distilled water. The concentrations of cadmium (Cd), iron (Fe), zinc (Zn), and magnesium (Mg) in the digested leaf and root samples were measured using inductively coupled plasma mass spectrometry (ICP-MS; Thermo Scientific, USA).

### Statistical analysis

Data collected on growth parameters, physiological responses, and cadmium accumulation were analyzed using two-way analysis of variance (ANOVA) to evaluate the effects of *Acinetobacter schindleri* SR-5-1 inoculation, Cd stress, and their interaction. Block effects were included in the model to account for variability among replicates. Before analysis, the data were tested for normality and homogeneity of variances using Shapiro-Wilk and Levene’s tests, respectively. Significant differences among treatment means were identified using Tukey’s Honestly Significant Difference (HSD) test at a 95% confidence level (*p* < 0.05). The results are presented as means  ±  standard error (SE) based on four replicates. Graphical representations of the data include error bars and statistical annotations to clearly indicate significant differences between treatments. Additionally, correlation analyses were performed to explore the relationships between growth, physiological traits, and cadmium accumulation, providing further insight into the role of *A. schindleri* SR-5-1 in mitigating cadmium toxicity. All the statistical analyses were conducted using Minitab (version 21.2), and graphical visualizations were prepared using Origin Pro 2021.

## Results

### Impact of *A. schindleri* SR-5-1 on growth parameters of pea under Cd stress

**Figure 1.**
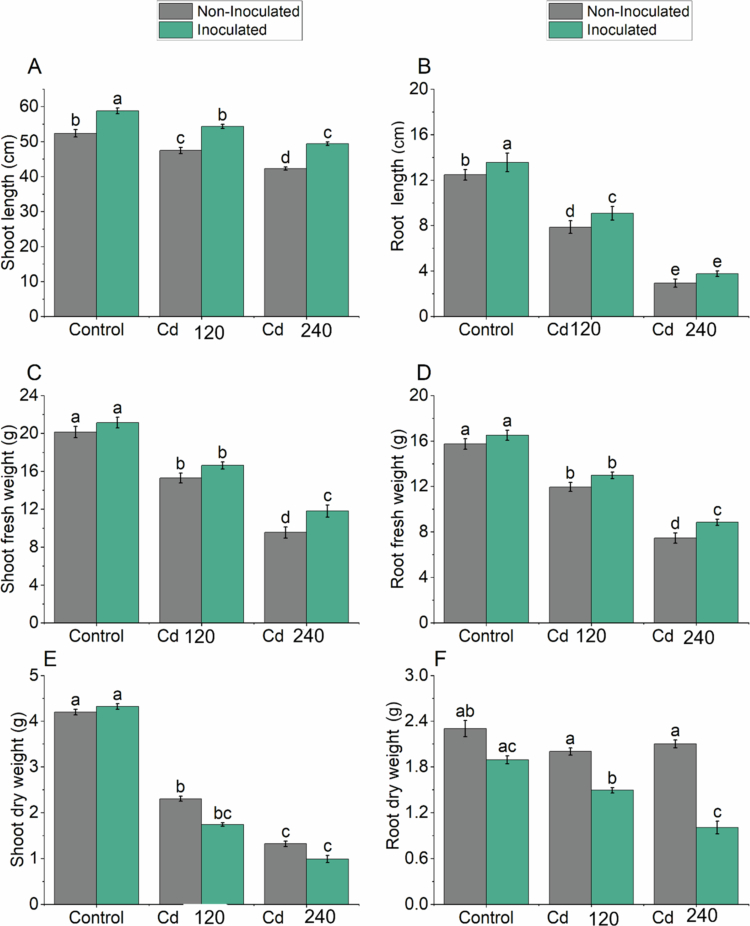
Effect of *Acinetobacter schindleri* SR-5-1 on (A) shoot length, (B) root length, (C) shoot fresh weight, (D) root fresh weight, (E) shoot dry weight, and (F) root dry weight under cadmium (Cd) toxicity at concentrations 120 and 240 mg/kg in pea (*Pisum sativum* L.) plants. Lowercase letters indicate significant differences according to Tukey’s test at *P* < 0.01.

Exposure to Cd at concentrations of 120 and 240 mg/kg caused a marked (*p* ≤ 0.001) reduction in shoot length (SL) and root length (RL), with decreases of approximately 10.39% and 58.47% at 120 mg/kg Cd, and more severe reductions of 23.86% and 324.4% at 500 mg/kg Cd, respectively. The inoculation of *A. schindleri* SR‑5‑1 had a significant beneficial role in the growth of pea plants under Cd stress. More importantly, inoculation mitigated the inhibitory effects of Cd; at 120 mg/kg Cd, inoculated plants experienced improvements in SL and RL (12.67% and 21.56%, respectively), while at 240 mg/kg Cd, these reductions were limited to 14.37% and 29.46% improvement, respectively. Similarly, shoot fresh weight (SFW) and root fresh weight (RFW) were significantly diminished by Cd stress, with declines of 31.69% and 32.1% at 250 µM, and 111.1% and 112% at 240 mg/kg Cd, respectively. SR‑5‑1 inoculation enhanced SFW and RFW in stressed plants by 8.01% and 9.1% under 250 µM Cd stress, and by 19.19% and 18.24% at 240 mg/kg Cd. In terms of dry biomass, shoot dry weight (SDW) and root dry weight (RDW) declined substantially with increasing Cd concentration (31.68% for shoots and 42.36% for roots). Although SR‑5‑1 inoculation improved fresh biomass, the dry biomass response was less consistent, with only marginal or non‑significant changes compared to uninoculated controls (*p* > 0.05) (Figure 1C–F). Overall, the interaction between Cd stress and SR‑5‑1 inoculation was statistically significant (*p* ≤ 0.001), highlighting the ability of the bacterium to alleviate Cd toxicity and promote growth, primarily by enhancing water status and fresh biomass accumulation under Cd stress conditions (Figure 1).

### Impact of *A. schindleri* SR-5-1 on photosynthetic pigments of Cd-stressed pea

**Figure 2.**
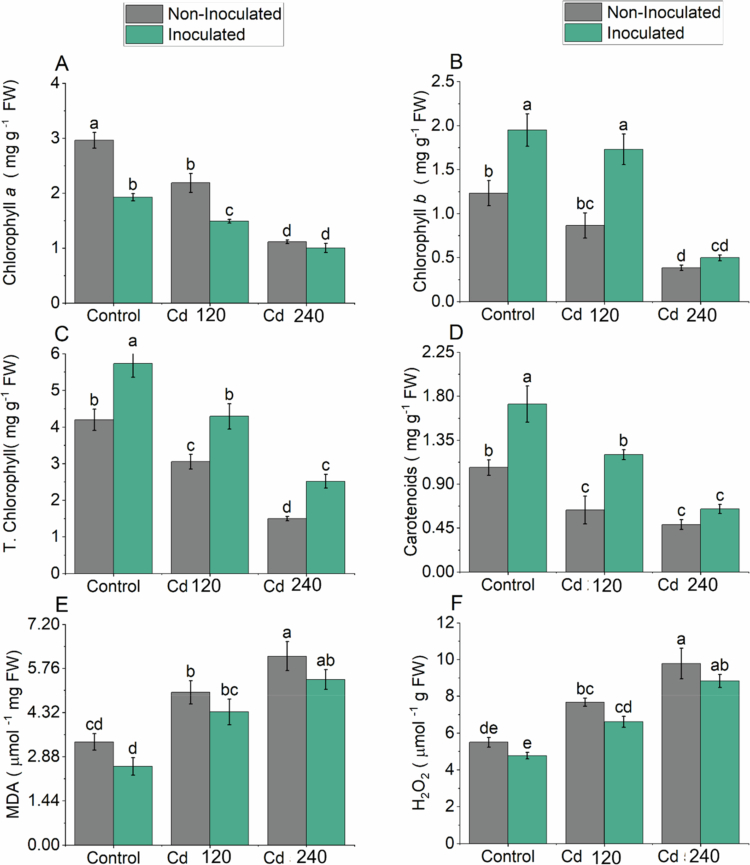
Effect of *Acinetobacter schindleri* SR-5-1 on (A) chlorophyll a, (B) chlorophyll b, (C) total chlorophyll, (D) carotenoids, (E) malondialdehyde (MDA), and (F) hydrogen peroxide (H₂O₂) under cadmium (Cd) toxicity at concentrations of 120 and 240 mg/kg in pea (*Pisum sativum* L.) plants. Lowercase letters indicate significant differences according to Tukey’s test at *P* < 0.01.

Cadmium at doses of 250 and 500  µM significantly (*p* ≤ 0.001) reduced the photosynthetic pigments of pea plants. Chlorophyll *a* (Chl *a*) levels decreased by 35.51% at 250 µM and 165.35% at 500 µM, while chlorophyll *b* (Chl *b*) levels decreased by 42.65% and 221.11%, respectively, compared to untreated controls. The total chlorophyll content also declined significantly, with losses of 37.54% and 180.27% at the two Cd doses. Carotenoids, which protect the photosynthetic machinery, decreased by 68.64% at 250 µM and 120.2% at 240 mg/kg Cd. However, inoculation with *A. schindleri* SR-5-1 mitigated these detrimental impacts. Under 120 mg/kg Cd stress, inoculated plants showed improvements of 46.89% in Chl *a*, 50.06% in Chl *b*, 28.13% in total chlorophyll, and 47.20% in carotenoids. At 240  mg/kg Cd, the recovery was more pronounced, with increases of 10.93%, 22.75%, 40.53%, and 24.61% in Chl *a*, Chl *b*, total chlorophyll, and carotenoids, respectively. This study found a strong relationship between cadmium stress and bacterial inoculation (p ≤ 0.001), indicating that SR-5-1 can protect and preserve photosynthetic performance in pea plants subjected to Cd toxicity (Figure 2).

Pea plants exposed to Cd at concentrations of 120 and 240  mg/kg Cd significantly (*p* ≤ 0.001) augmented oxidative stress markers. Malondialdehyde (MDA) content rose by 32.43% at 250 µM and 45.5% at 500 µM compared to untreated controls. Similarly, hydrogen peroxide (H₂O₂) levels increased by 28.39% and 43.82% at the respective Cd concentrations. Inoculation with *A. schindleri* SR-5-1 substantially mitigated these harmful effects. Under 120 mg/kg stress, inoculated plants exhibited decreases in MDA by 14.59% and H₂O₂ by 16.13%, while under 240 mg/kg Cd, reductions of 14.21% in MDA and 10.80% in H₂O₂ were observed. Although the interaction between cadmium exposure and bacterial inoculation was not statistically significant (*p* > 0.05), the consistent downward trend demonstrated that SR-5-1 alleviated oxidative damage and supports stress tolerance (Figure 2).

### Enzymatic antioxidants of pea under Cd stress and *A. schindleri* SR-5-1 inoculation

**Figure 3.**
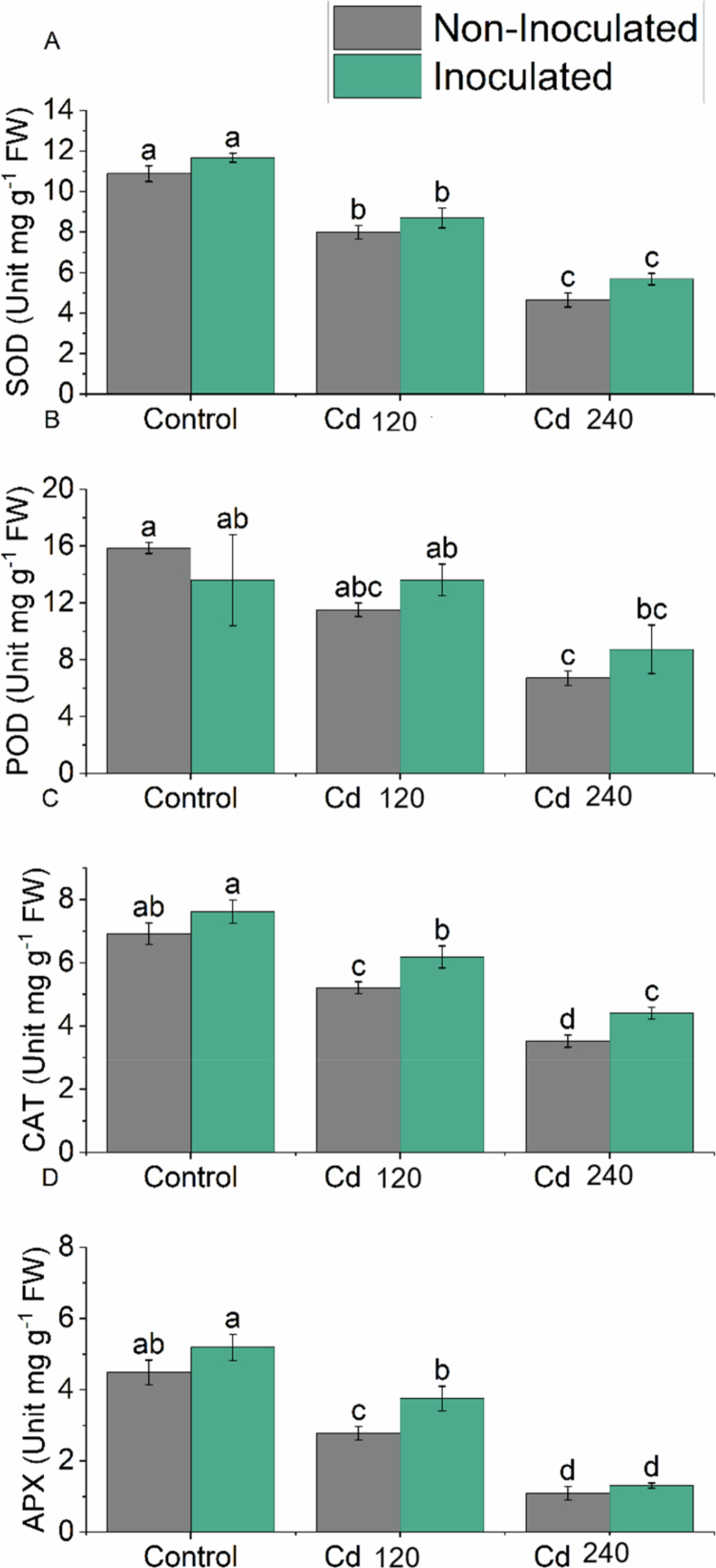
Effects of *Acinetobacter schindleri* SR-5-1 on (A) superoxide dismutase (SOD), (B) peroxidase (POD), (C) catalase (CAT), and (D) ascorbate peroxidase (APX) under cadmium (Cd) toxicity at concentrations of 120 and 240 mg/kg in pea (*Pisum sativum* L.) plants. Lowercase letters indicate significant differences according to Tukey’s test at *P* < 0.01.

Pea plants subjected to Cd (120 and 240  mg/kg Cd) exhibited a marked (*p* ≤ 0.001) increase in oxidative stress, accompanied by significant suppression of key antioxidant enzyme activities. Superoxide dismutase (SOD) decreased by 36.09%, peroxidase (POD) by 37.80%, catalase (CAT) by 32.87%, and ascorbate peroxidase (APX) by 61.64% under Cd stress compared to controls. Inoculation with *A. schindleri* SR-5-1 substantially mitigated these adverse effects. Under Cd stress at 120 and 240 mg/kg Cd, inoculated plants showed increases in SOD (7.99% and 18.10%), POD (15.45% and 23.26%), CAT (16.35% and 20.13%), and APX (25.98% and 16.53%), respectively, thereby restoring antioxidant enzyme activities. Although the interaction between Cd exposure and bacterial inoculation was not statistically significant (*p* > 0.05), the consistent upward trend demonstrated that SR-5-1 alleviated Cd-induced oxidative damage by enhancing antioxidant defences (Figure 3).

### Impact of *A. schindleri* SR-5-1 inoculation on the nutrient profile of pea under Cd stress

**Figure 4.**
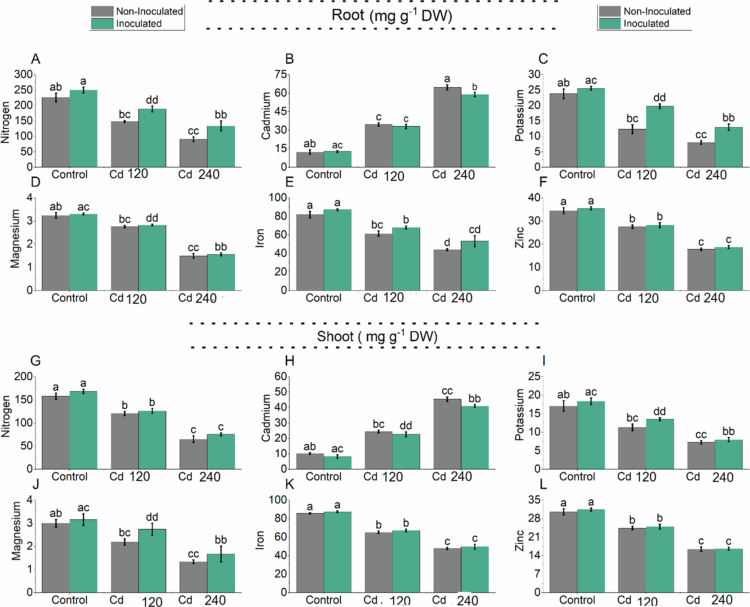
Effect of *Acinetobacter schindleri* SR-5-1 on the root content of (A) nitrogen, (B) cadmium, (C) potassium, (D) magnesium, (E) iron, and (F) zinc, and the shoot content of (G) nitrogen, (H) cadmium, (I) potassium, (J) magnesium, (K) iron, and (L) zinc under cadmium (Cd) toxicity at concentrations of 120 and 240  mg/kg in pea (*Pisum sativum* L.) plants. Lowercase letters indicate significant differences according to Tukey’s test at *P* < 0.01.

Exposure of pea plants to Cd stress at both 120 and 240  mg/kg Cd concentrations resulted in a marked reduction (*p* ≤ 0.001) in essential nutrient contents, namely, nitrogen (N), iron (Fe), zinc (Zn), potassium (K), and magnesium (Mg) in both leaves and roots compared to untreated controls. At 120  mg/kg Cd, the leaf N, K, Mg, Fe, and Zn contents decreased by 31.11%, 51.21%, 36.90%, 31.34%, and 25.18%, respectively, while root concentrations of these nutrients declined by 52.47%, 92.97%, 17.39%, 33.42%, and 25.18%, respectively. The detrimental impact was more pronounced at 240  mg/kg Cd, where the leaf N, Fe, Zn, K, and Mg concentrations were reduced by 141.6%, 135.31%, 123.28%, 79.66%, and 85.82%, respectively, and the root concentrations were decreased by 148.89%, 197.5%, 116.96%, 85.30%, and 93.87%, respectively, compared to the control. Inoculation with *A. schindleri* SR-5-1 substantially improved nutrient absorption and accumulation under Cd stress (*p* ≤ 0.001). This bacterial inoculation alleviated the adverse impact of Cd toxicity by enhancing nutrient availability and uptake, thereby supporting better plant nutrition and growth under Cd-contaminated conditions. Although the interaction between Cd exposure and bacterial inoculation was not statistically significant (*p* > 0.05), the consistent improvement demonstrated that SR-5-1 enhanced nutrient acquisition under heavy metal stress (Figure 4).

### Cadmium accumulation in pea grown with *A. schindleri* SR-5-1

The cadmium content in pea plants significantly (*p* ≤ 0.001) increased in both roots and leaves when supplied at 120 and 240 mg/kg Cd concentrations compared to untreated controls. At 120 Cd, Cd accumulation in the leaves and roots increased by approximately 58.10% and 64.42%, respectively, while at 240 Cd, the accumulation further increased by approximately 77.59% in the leaves and 80.98% in the roots. However, seed inoculation with *A. schindleri* SR-5-1 markedly reduced the Cd content in both tissues under Cd stress. The inoculated pea plants showed decreases in the leaf Cd concentration of approximately 7.21% and 10.85% at 120 and 240 Cd, respectively, while root Cd content was reduced by approximately 4.48% and 9.67%, respectively, at the same concentrations. These reductions demonstrate the effectiveness of *A. schindleri* SR-5-1 in limiting Cd uptake and translocation within pea plants, thereby mitigating Cd toxicity and enhancing plant tolerance under contaminated conditions. Although the interaction between Cd exposure and bacterial inoculation was not statistically significant (*p* > 0.05), the consistent downward trend confirms that SR-5-1 alleviates Cd accumulation (Figure 4).

#### Histochemical

**Figure 5.**
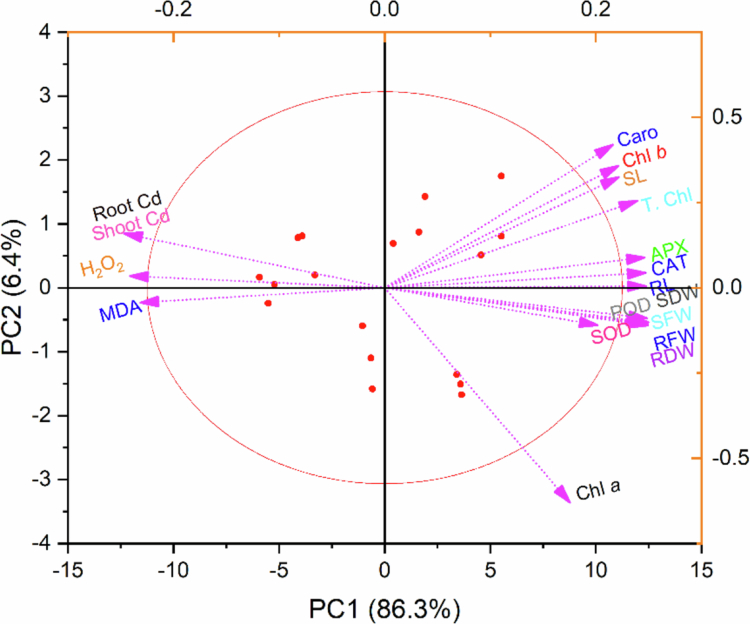
Principal component analysis revealed that cadmium (Cd) accumulation in plants is negatively correlated with growth, chlorophyll pigments, and enzymatic antioxidant compounds.

Histochemical staining showed that cadmium stress led to strong ROS accumulation and lipid peroxidation in non-inoculated pea plants, evident from intense brown (DAB), blue (NBT), and magenta (Schiff’s reagent) deposits. In contrast, plants inoculated with *Acinetobacter schindleri* SR-5-1 exhibited much weaker staining, indicating reduced hydrogen peroxide, superoxide, and membrane damage. Overall, the results confirmed that bacterial inoculation effectively alleviated oxidative stress and protected leaf tissues under cadmium exposure (Figure 5).

## Discussion

**Figure 6.**
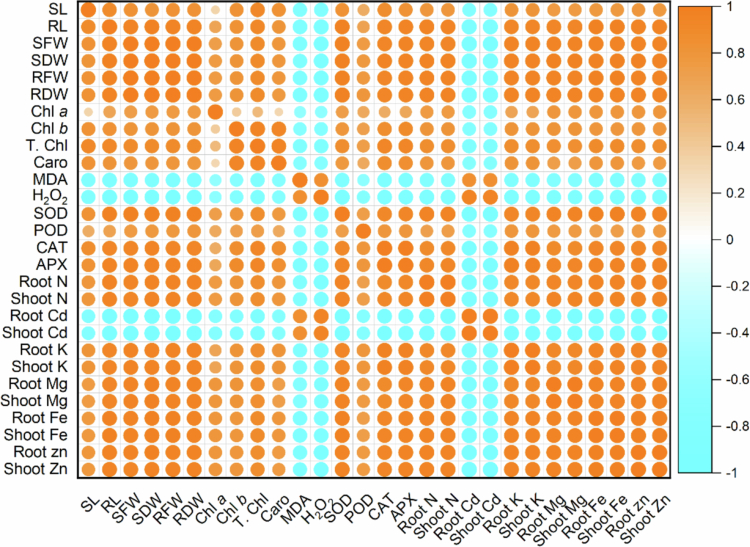
Pearson correlation analysis of physiological, biochemical, and nutrient variables. Abbreviations: shoot length (SL), root length (RL), shoot fresh weight (SFW), shoot dry weight (SDW), root fresh weight (RFW), root dry weight (RDW), chlorophyll a (Chl a), chlorophyll b (Chl b), total chlorophyll (T. Chl), carotenoids (Caro), malondialdehyde (MDA), hydrogen peroxide (H₂O₂), superoxide dismutase (SOD), peroxidase (POD), catalase (CAT), ascorbate peroxidase (APX), root nitrogen (Root *N*), shoot nitrogen (Shoot *N*), root cadmium (Root Cd), shoot cadmium (Shoot Cd), root potassium (Root K), shoot potassium (Shoot K), root magnesium (Root Mg), shoot magnesium (Shoot Mg), root iron (Root Fe), shoot iron (Shoot Fe), root zinc (Root Zn), and shoot zinc (Shoot Zn).

Cadmium toxicity poses significant challenges to the biomass of pea plants through physiological disruption. This metal hampers root seedlings and the formation of lateral roots by interfering with the activity of meristematic tissues and cell division. As a result, the plants struggle to absorb essential nutrients and water. In the above-ground parts of the plant, cadmium interferes with the production of chlorophyll and damages Photosystem II, leading to a marked decline in photosynthetic efficiency and carbon uptake. The consequences of these metabolic impairments manifest as stunted growth, lower biomass accumulation, and reduced yield. Additionally, cadmium induces oxidative stress by increasing the excess ROS. This imbalance results in lipid peroxidation and degradation of cellular membranes, undermining the structural integrity and overall physiological functions of the plant. To complicate matters further, cadmium competes with vital elements such as zinc, iron, and magnesium at transport sites, leading to nutritional deficiencies that further exacerbate the inhibition of development in pea plants. Inoculation with *Acinetobacter schindleri* SR-5-1 counteracts these deleterious effects through several synergistic mechanisms. The bacterium enhances nutrient availability via siderophore-mediated iron solubilization and phosphate mineralization, improving the nitrogen, potassium, and magnesium acquisition critical for chlorophyll synthesis and enzyme activation. By producing ACC deaminase, SR-5-1 downregulates stress-induced ethylene accumulation, preserving root architecture and promoting lateral root development for better resource foraging. Studies corroborate these mechanisms. Tanwir et al.^45^ demonstrated SR-5-1's efficacy in enhancing pea growth under Cd stress through improved antioxidant defence and reduced metal translocation. Similarly, Bacillus strains exhibiting analogous plant growth-promoting traits mitigated Cd toxicity in crops by modulating nutrient uptake and stress hormone levels. The bacterium's ability to retain functionality across contamination gradients (120 and 240 mg/kg Cd) underscores its utility as a bioinoculant for sustainable legume cultivation in polluted agroecosystems. Schematic representation of pea plants responding to Cd stress with and without inoculant, as depicted in Figure 6.

**Figure 7.**
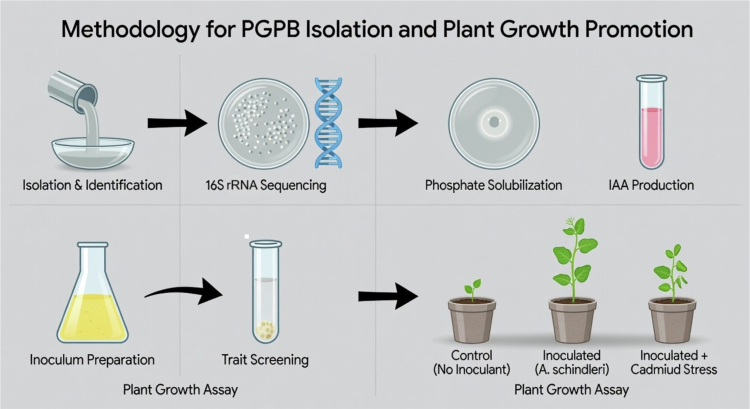
Schematic diagram showing that inoculation with *Acinetobacter schindleri* SR-5-1 enhanced growth under cadmium (Cd; 120 and 240  mg/kg) toxicity in pea plants.

The process of chlorophyll defoliation in pea plants administered with cadmium led to diminished overall plant growth and development. Cadmium ions exhibit a propensity to interact with chlorophyll molecules by substituting the central magnesium ion (Mg²⁺) within their molecular structure. This substitution results in the development of a Cd‒chlorophyll complex, which destabilizes the pigment and modifies its light-absorbing properties, thereby leading to a reduction in photosynthetic efficiency.^46^ Additionally, cadmium demonstrates inhibitory effects on key enzymes that are critical for chlorophyll biosynthesis, including protochlorophyllide reductase and various enzymes involved in the synthesis of 5-aminolevulinic acid, a pivotal precursor in the chlorophyll biosynthetic pathway. Such enzymatic inhibition not only diminishes the rate of chlorophyll synthesis but also accelerates the decomposition of the pigment.^47^ *Acinetobacter schindleri* SR-5-1 reduces cadmium (Cd) toxicity in plants by activating various complementary processes that reduce Cd accumulation and ameliorate its adverse effects, improving chlorophyll content and overall plant development. This strain promotes development and reduces oxidative stress indicators (MDA and H_2_O_2_) in pea and other crops under Cd stress by restricting Cd absorption and boosting nutrient acquisition. One significant method is bacterial sequestration and immobilization of Cd ions in the rhizosphere by biosorption and binding to bacterial cell walls and exopolysaccharides, hence limiting Cd bioavailability to plant roots. This reduces Cd translocation to shoots and chloroplasts, so preserving the photosynthetic apparatus from Cd-induced damage and chlorophyll degradation. Other crops confirm similar protective roles of Acinetobacter species. For example, the inoculation of Eichhornia crassipes with Acinetobacter strains enhanced copper phytoremediation by increasing metal sequestration in roots and reducing shoot accumulation, thereby protecting photosynthetic tissues. The bacterial transformation of Cd into less toxic forms, such as cadmium sulfide nanoparticles has also been documented, representing an additional detoxification route. Collectively, these mechanisms enable *Acinetobacter schindleri* SR-5-1 to mitigate Cd by decreasing Cd absorption, enhancing nutrient status, and protecting chlorophyll from degradation, ultimately improving plant growth and productivity in polluted environments. Pearson correlation coefficients were computed among all measured parameters, and their significance was tested (two-tailed, *p* < 0.05, *p* < 0.01, *p* < 0.001). Only significant correlations are emphasized in Figure 7, while non-significant relationships are shown with reduced intensity.

**Figure 8.**
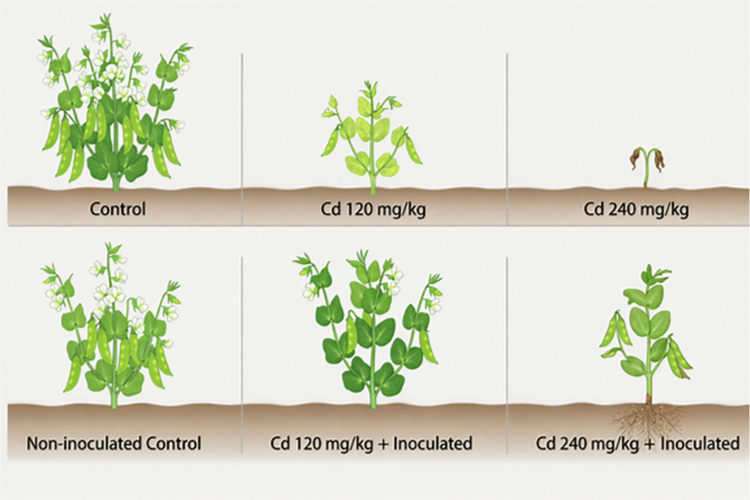
Effect of *Acinetobacter schindleri* SR-5-1 inoculation on pea plant growth under cadmium stress.

Morphological observations revealed that cadmium stress severely inhibited pea plant growth in non-inoculated treatments, as evidenced by reduced plant height. In contrast, plants inoculated with *Acinetobacter schindleri* SR-5-1 maintained better vigor, had greener foliage, and improved reproductive development even under 120 and 240  mg/kg Cd exposure. The visual comparison clearly demonstrated the protective role of bacterial inoculation in sustaining plant morphology and overall growth performance under cadmium toxicity (Figure 8).

**Figure 9.**
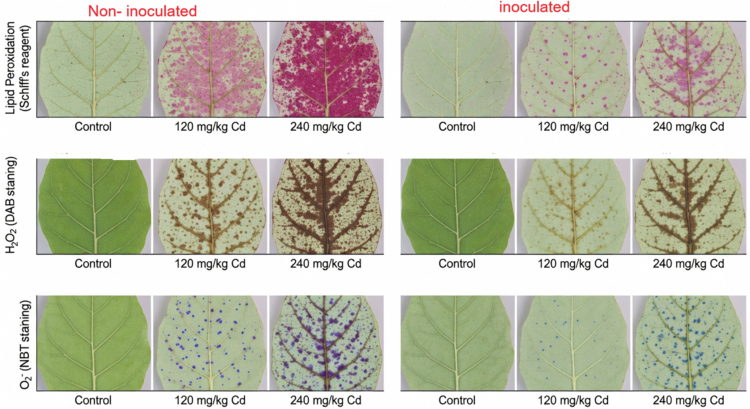
Histochemical detection of oxidative stress markers in pea leaves under cadmium exposure with or without Acinetobacter schindleri SR-5-1 inoculation. Leaves were stained using Schiff’s reagent for lipid peroxidation, DAB for hydrogen peroxide (H₂O₂), and NBT for superoxide (O₂⁻) accumulation. Increased staining intensity at higher Cd concentrations indicates elevated ROS generation, while inoculated plants exhibited reduced oxidative damage compared to non-inoculated controls.

Cadmium (Cd) stress disrupts the activities of key antioxidant enzymes, such as SOD, POD, CAT, and APX, through several mechanistic pathways. Although Cd itself is not a redox-active metal and cannot directly generate ROS, it indirectly induces oxidative stress by interfering with cellular metabolism and organelle function, particularly in chloroplasts and mitochondria. This interference leads to excessive ROS production, which damages proteins, lipids, and nucleic acids, impairing enzyme structure and function. The principal component analysis showed that Cd content was negatively associated with nutrient ions (Figure 7). One mechanism behind the decreased activities of SOD, POD, CAT, and APX under Cd stress is the direct binding of Cd ions to the active sites or sulfhydryl groups of these enzymes, causing conformational changes and enzyme inactivation. Cd can displace essential metal cofactors such as zinc, copper, manganese, and iron, which are critical for the catalytic activity of these antioxidant enzymes, leading to their reduced efficiency. Additionally, Cd-induced nutrient imbalances, especially deficiencies of Fe and Mn, further compromise enzyme synthesis and activity. Several studies have reported these effects; for example, in *Phaseolus vulgaris*, Cd exposure led to decreased antioxidant enzyme activities due to Cd accumulation in roots and disruption of systemic signaling.^48,49^ In *Cucurbita pepo*, Cd stress inhibited growth and reduced catalase and superoxide dismutase activities, reflecting impaired antioxidant defence.^50^ Similarly, in soybean, Cd toxicity altered enzyme activities by interfering with cofactor availability and enzyme stability.^51,52^ *A. schindleri* SR-5-1 enhances the plant’s antioxidant defence system under cadmium (Cd) stress, possibly by multiple mechanisms, such as IAA synthesis and ACC deaminase activity, since the strain has been previously reported to have these attributes.^32^ This bacterium stimulates the activities of key antioxidant enzymes, such as SOD, CAT, POD, and APX, which play important roles in scavenging ROS and reducing oxidative damage in plants exposed to Cd. By enhancing these enzymatic defences, A. *schindleri* SR-5-1 helps maintain the cellular redox balance and protects chloroplasts and other organelles from Cd-induced injury. Additionally, *A. schindleri* SR-5-1 reduces Cd bioavailability in the rhizosphere through biosorption and metal sequestration, limiting Cd absorption by the roots and subsequent aerial parts of plants. SR-5-1 inoculation restored antioxidant enzyme activities and reduced the expression of oxidative stress markers, indicating microbial regulation of ROS detoxification pathways and improved resilience under Cd toxicity. Principal component analysis revealed that cadmium (Cd) accumulation in plants is negatively correlated with growth, chlorophyll pigments, and enzymatic antioxidant compounds (Figure 9).

Cadmium (Cd) stress decreases nutrient accumulation in plants through several interconnected mechanisms. Firstly, Cd competes with essential mineral nutrients such as calcium (Ca), magnesium (Mg), iron (Fe), zinc (Zn), and manganese (Mn) for uptake sites and transporters in the root system. This competition reduces the availability and absorption of these nutrients, leading to deficiencies that impair plant metabolism and growth. Cd can enter plant roots via channels normally used for Ca ions, disrupting ion homeostasis and causing nutrient imbalances. Cd alters plasma membrane permeability and damages membrane integrity, resulting in the leakage of nutrients from root cells and impaired selective transport across membranes. This disruption affects both absorption and movement in pea plants. Additionally, Cd stress inhibits stomatal opening by disturbing the plant’s water balance, reducing transpiration-driven nutrient flow and further limiting nutrient transport to aerial parts. Cd interferes with photosynthesis by inhibiting key enzymes of the Calvin cycle and electron transport chain, which decreases the plant’s energy supply necessary for active nutrient uptake and assimilation. The combined effect of reduced nutrient uptake and impaired metabolic processes leads to lower concentrations of macro- and micronutrients in both roots and shoots. Cd induces changes in root architecture and biomass allocation, often causing plants to prioritize root growth overshoots to acquire more nutrients from the soil, but this is usually insufficient to compensate for the overall nutrient deficiency caused by Cd toxicity. *A. schindleri* SR-5-1 has been shown to significantly alleviate Cd toxicity and improve growth and nutrient acquisition in various crops, including pea (*Pisum sativum*) and linseed (*Linum usitatissimum*).^32^ Although Cd and essential cations share common uptake pathways, SR-5-1 inoculation enhances nutrient acquisition by selectively favoring Zn, Fe, K, and Mg uptake while restricting Cd transport, ensuring better nutritional homeostasis under stress.

Under Cd and wastewater stress, both pea and linseed exhibit reduced growth, biomass, antioxidant capacity, and nutrient uptake due to increased oxidative damage and Cd accumulation.^32^ However, inoculation with A. schindleri SR-5-1 markedly enhances morpho-physiological and biochemical traits by reducing malondialdehyde (MDA) and hydrogen peroxide (H_2_O_2_) levels, which are indicators of oxidative stress. This bacterial inoculation also lowered Cd uptake in roots and shoots while maintaining optimal levels of essential nutrients such as nitrogen, phosphorus, potassium, iron, and magnesium, thereby promoting better growth and metabolism under stress conditions.^53^ The protective effects of *Acinetobacter* species have been reported in other crops. For example, inoculation of maize cultivars with Acinetobacter sp. SG-5 improved growth, antioxidant enzyme activities, and nutrient acquisition under Cd stress, particularly in Cd-sensitive cultivars. The bacteria enhance phytohormonal balance, reduced Cd uptake, and mitigated oxidative damage, resulting in improved biomass and photosynthetic pigment content.^33^, ^54^ In *Eichhornia crassipes*, *Acinetobacter* strains increased heavy metal phytoremediation efficiency by promoting metal accumulation in roots and reducing translocation to shoots, which protected photosynthetic tissues and maintained nutrient balance (Kurniawan et al., 2017). *A. schindleri* SR-5-1 might have alleviated Cd toxicity through multiple pathways, including its strong potential to produce ACC deaminase. It produces siderophores that chelate iron and other micronutrients, enhancing their availability and uptake by plants, which is critical under Cd-induced nutrient deficiencies. The bacterium also secretes exopolysaccharides that immobilize Cd ions in the rhizosphere, reducing their bioavailability and uptake. Moreover, *A. schindleri* possesses efficient Cd efflux systems, such as cation diffusion facilitators and heavy metal efflux pumps, which help detoxify Cd in the soil environment and limit its accumulation in plant tissues. The bacterium’s production of ACC deaminase lowers ethylene levels in plants, mitigating stress-induced growth inhibition and preserving root development. Additionally, *A. schindleri* enhances antioxidant enzyme activities (SOD, CAT, POD, and APX), protecting plants from oxidative harm, as shown by Cd.^53,54^ SR-5-1 inoculation significantly decreased Cd accumulation in pea tissues, likely through rhizosphere immobilization and selective modulation of transporter activity, thereby reducing Cd bioavailability and translocation.

The cadmium (Cd) content in pea plants increases in both roots and leaves when exposed to Cd stress, primarily due to the high bioavailability of the metal and its ability to enter plant roots via transporters that normally uptake essential divalent cation minerals. Once inside the root cells, Cd is taken up by binding to cell wall components and intracellular ligands, limiting its detoxification and causing its translocation to aerial parts through the xylem. The increased Cd concentration in roots and leaves disrupts physiological processes, inhibits nutrient uptake, and induces toxicity symptoms.^55^ *A. schindleri* SR-5-1 enhances plant antioxidant defences by stimulating enzymes such as superoxide dismutase, catalase, and peroxidases, which protect plants from Cd-induced oxidative damage, indirectly supporting nutrient uptake and growth.^54^ The ACC deaminase activity of bacteria reduces ethylene levels in plants under stress, promoting root growth and improving nutrient acquisition. Collectively, these mechanisms limit Cd movement from roots to shoots, reduce Cd toxicity, and maintain essential nutrient homeostasis, resulting in improved plant growth and productivity under Cd stress.^55^ Cadmium absorption in plants under stress results from its uptake via divalent cation transporters and limited detoxification capacity, leading to increased metal content in roots and leaves. *A. schindleri* SR-5-1 mitigates this effect by actively removing Cd from the rhizosphere through efflux pumps, immobilizing Cd via exopolysaccharides and siderophores, transforming Cd into less toxic forms, and enhancing plant antioxidant and growth-promoting responses, thereby reducing Cd uptake and toxicity.

Cadmium and other divalent cations (Zn²⁺, Fe²⁺, Mg²⁺, etc.) indeed share common uptake systems in plants, primarily through broad-spectrum transporters such as the ZIP, NRAMP, and HMA families. The reduction in Cd accumulation under *Acinetobacter schindleri* SR-5-1 treatment, despite enhanced uptake of essential cations, can be explained by the selective modulation of transporter activity and rhizosphere detoxification. SR-5-1 likely immobilizes Cd in the rhizosphere through biosorption and chelation, thereby reducing its bioavailability. At the same time, microbial inoculation improves root physiology and signaling, enhancing the efficiency of nutrient transporters for beneficial cations. Thus, the observed pattern reflects microbial-driven discrimination between toxic and essential ions rather than a simple competitive uptake process.

## Conclusion

This field study comprehensively evaluated the effects of Cd stress at concentrations of 120 and 240 mg/kg on pea (*Pisum sativum* L.) plants and the mitigating effects of inoculation with *Acinetobacter schindleri* SR-5-1 under realistic agronomic conditions. The results clearly demonstrated that Cd stress meaningfully impaired plant growth by reducing plant biomass and chlorophyll content. These detrimental effects were accompanied by increased oxidative stress, as evidenced by elevated malondialdehyde (MDA) and hydrogen peroxide (H_2_O_2_) levels, and a decline in antioxidant enzyme activities (SOD, POD, CAT, and APX). Furthermore, Cd exposure disrupted nutrient uptake, causing deficiencies in essential macro- and micronutrients such as nitrogen, iron, zinc, potassium, and magnesium, while significantly increasing Cd accumulation in both roots and leaves, thereby exacerbating toxicity in plants. Importantly, inoculation with *Acinetobacter schindleri* SR-5-1 substantially alleviated Cd-induced toxicity by enhancing growth parameters and restoring the chlorophyll content, indicating improved photosynthetic efficiency and biomass production. The bacterium effectively reduced oxidative damage by upregulating the expression of antioxidant enzymes, thereby protecting cellular structures and maintaining physiological functions under Cd stress. Additionally, *A. schindleri* SR-5-1 improved nutrient acquisition and homeostasis, mitigating Cd-induced nutrient imbalances. These findings validate the efficacy of *A. schindleri* SR-5-1 as a potent bioinoculant for enhancing plant tolerance to Cd stress under field conditions, bridging the gap between controlled pot studies and practical agricultural applications. This study emphasizes the importance of integrating beneficial microbial inoculants into sustainable crop management strategies to improve crop productivity and food safety in Cd-contaminated soils. Future research should focus on long-term field trials across diverse soil types and environmental conditions to further optimize microbial-assisted remediation approaches and facilitate their large-scale adoption in heavy metal-affected agroecosystems.
